# Positive effect of timed blue-enriched white light on sleep and cognition in patients with mild and moderate Alzheimer’s disease

**DOI:** 10.1038/s41598-021-89521-9

**Published:** 2021-05-13

**Authors:** Seong Jae Kim, Sun Hee Lee, In Bum Suh, Jae-Won Jang, Jin Hyeong Jhoo, Jung Hie Lee

**Affiliations:** 1 Department of Psychiatry, Cheongju Hospital, Cheongju, South Korea; 2 Department of Psychiatry, Silverheals Hospital, Namyangju, South Korea; 3 Department of Laboratory Medicine, Kangwon National University Hospital, Chuncheon, South Korea; 4 Department of Neurology, Kangwon National University Hospital, Chuncheon, South Korea; 5 Department of Neurology, Kangwon National University School of Medicine, Chuncheon, South Korea; 6 Department of Psychiatry, Kangwon National University Hospital, Chuncheon, South Korea; 7 Department of Psychiatry, Kangwon National University School of Medicine, Chuncheon, South Korea

**Keywords:** Diseases, Medical research, Neurology

## Abstract

Conflicting results have been reported regarding the effectiveness of light treatment (LT) in patients with Alzheimer’s disease (AD). We investigated the effectiveness of blue-enriched white LT on sleep, cognition, mood and behavior in patients with mild and moderate AD. The treatment group (n = 14) sat about 60 cm away from a small (136 × 73 × 16 mm) LED light box for 1 h each morning for 2 weeks. The control group (n = 11) wore dark, blue-attenuating sunglasses during the 1 h exposures. The morning light started 9–10 h after each individual’s dim light melatonin onset (DLMO). Assessments were done at baseline (T0), immediate post-treatment (T1), and 4 weeks after the end of the 2 weeks of LT (T2). Sleep was measured by actigraphy. Blue-enriched LT had a significantly better effect on the Pittsburgh Sleep Quality Index at T2 compared to blue-attenuated LT, and a trend of better effectiveness on total sleep time at T2. There was a significant increase in Mini-Mental State Examination score at T2 after blue-enriched LT than that at T0. Our findings suggest that morning blue-enriched LT has a benefit in improving sleep and cognitive function in AD patients.

## Introduction

Sleep disturbances are prevalent in patients with Alzheimer’s disease (AD). Approximately 45% of AD patients have sleep problems^1^. Sleep disturbances are characterized by symptoms of fragmented sleep, night–day reversal, disrupted sleep–wake rhythms, and so on. This has been known to increase caregiver burden and the risk of institutionalization^2,3^.

It has been reported that sleep disturbance is associated with cognitive dysfunction of AD patients^4^. Besides, AD patients who are experiencing sleep disturbances are likely to suffer from mood and/or behavioral symptoms concurrently. A study on patients with mild and moderate AD has reported the association of sleep disturbance with behavioral symptoms, notably aggressiveness^5^. There are some evidences showing a significant loss in suprachiasmatic nucleus (SCN) of AD patients that is correlated with diminished sleep–wake rhythmicity^6^. To some extent, underlying circadian disturbances in AD patients could be responsible for their sleep and behavioral disturbances. In addition, insufficient light exposure which might be a cause of sleep and behavior disturbances can be problematic, particularly in institutionalized patients^7^.

As the light is a central modulator of circadian rhythms, therapeutic exposure of AD patients to bright light is expected to improve their sleep mainly by stabilizing their sleep–wake rhythms^8–11^. Besides, bright light treatment (bright LT) can improve the mood and cognitive function of AD patients by stabilizing their circadian rhythm or leading to neural stimulation of brain regions involved in alertness, emotion, and cognition^12,13^. It has been generally shown that both morning and evening bright LTs have beneficial effects on sleep and behavior of AD patients only when bright LT with enough duration and intensity is given to them^14,15^. However, many studies, even those with a randomized controlled design, have found no definite effect of bright LT on patients with dementia, including those with AD^9,10^.

These conflicting findings might result from different timing of applied bright LT, implicating that some patients might have received the light exposure during a sensitive region of the phase response curve while others have not^9–11,16^. Thus, it is important to determine the timing of LT based on individual circadian phase for each patient in order to elucidate the therapeutic effect of LT on AD patients. Another reason for these conflicting findings could be that study subjects are less likely to have greater responses to light since most studies have been conducted on severely demented patients whose SCNs were to be more degenerated^8,17^.

Our circadian system is known to be more sensitive to short wavelength light. A blue-enriched white light has been reported to have saturation effects even with an intensity of 750 lx. Its therapeutic effects are comparable to those of standard LT for patients with seasonal affective disorder^18^. However, it remains unclear whether the administered blue-enriched white light can enhance sleep quality of AD patients, although some studies have reported improvement of subjective sleep quality after its intervention in the patients with AD and/or related dementia^2,16^.

Phase delay in circadian rhythms has been found in AD patients. It is apparently discrete from what is observed in normal aging^7,15,19^. In a perspective of normalizing circadian rhythms, phase advance caused by LT would be reasonable to investigate its therapeutic effects on AD patients. Meanwhile, in a previous study on healthy young subjects, advance portions of blue light phase response curve have been presented from 09:00 to 20:00 after dim light melatonin onset (DLMO)^20^, a well-known marker for estimating the circadian phase^21^. Sletten et al.^22^ have also reported similar findings in healthy elderly subjects. However, to the best of our knowledge, no study has reported the effectiveness of LT in dementia patients based on their endogenous circadian rhythm.

Thus, the objective of this study was to investigate the effectiveness of timed blue-enriched white light treatment (timed BLT) on sleep, cognition, mood, and behavior of patients with mild and moderate AD based on individual circadian phase using DLMO compared to the effectiveness of timed blue-attenuating LT as control. Our hypothesis was that timed BLT applied based on individual DLMO would have more effects on outcome measures including subjective and objective sleep quality, mood and behavior symptoms, and cognitive function than the control (blue—attenuating LT) immediately post-treatment and at 4-week follow-up.

## Methods

### Participants

Patients with mild and moderate Alzheimer’s disease (AD) who had a sleep disturbance and their caregivers were recruited from the Dementia Clinic at Kangwon National University Hospital, physician referrals, and advertisements between March 2017 and April 2018. The diagnosis of AD and its severity were determined by a psychiatrist or a neurologist using the Diagnostic and Statistical Manual of Mental Disorder, 5th Edition (DSM-5)^23^ and the Clinical Dementia Rating Scale (CDR)^24^, respectively. Patient eligibility criteria included a diagnosis of probable or possible AD for major neurocognitive disorder, a CDR score ranging from 0.5 to 2, a sleep disturbance verified by a score of 5 or greater on the Pittsburgh Sleep Quality Index (PSQI)^25^ and/or by one or more complaints among difficulty initiating sleep (DIS), difficulty maintaining sleep (DMS), and early morning awakening (EMA) for 3 or more days per week.

Patients were excluded if they met the following criteria: (1) current substance related disorders, depressive disorders, or other psychiatric disorders by DSM-5; (2) current medical illness including liver cirrhosis, chronic pulmonary disease, cancer, uncontrolled diabetes, and uncontrolled hypertension; (3) being suspected or diagnosed with primary sleep disorders except for insomnia disorder (i.e., restless legs syndrome, sleep-disordered breathing disorder; hypersomnia, or narcolepsy); (4) a prior history of cerebrovascular disease or central nervous system (CNS) disease, or evidence of CNS injury; (5) current use of any medication affecting their circadian rhythms (i.e., Circadin^R^); (6) changes in type or dose of taking hypnotics or CNS active drugs for the study period; (7) significant impairment of hearing ability, visual acuity, or language ability which hindered the completion of neurocognitive tests; (8) abnormalities in complete blood cell count, liver function test, urine analysis, electrocardiogram (ECG), or chest X-ray; and (9) pupillary abnormalities on a neuro-ophthalmological examination.

In a single-blind, enrolled patients were assigned into one of the treatment group (TG) and control group (CG) by turns, based on a constant allocation ratio of 1:1 if possible. Fourteen patients (77.36 ± 5.79 years; M:F = 2:12) in the TG and 11 patients (78.55 ± 7.71 years; M:F = 5:6) in the CG completed our study protocol.

The study protocol was approved by the Institutional Review Board of Kangwon National University Hospital following all relevant guidelines and regulations (Protocol ID: KNUH-2016-10-007-001). The study was registered with the clinical studies database Clinical Research Information Service(CRiS), Republic of Korea (KCT0005282, registration date: 08/04/2020; http://cris.nih.go.kr/cris/inc/login.jsp). Written informed consent was obtained from each participant and his/her legally authorized representatives prior to commencement of this study. All procedures were carried out in accordance with principles of the Declaration of Helsinki.

### Procedures

In the laboratory, Dim Light Melatonin Onset (DLMO) was determined from seven hourly saliva samples obtained starting from 6 h prior to the mean sleep onset measured by actigraphy (Actiwatch 2; Philips Respironics, Murrysville, PA, USA) recording for 5 days at baseline. Home-based 1-h timed BLT was applied between 9 and 10 h after DLMO for 2 weeks. The study protocol consisted of assessments of outcome and actigraphical measures at baseline (T0), immediate post-treatment (T1), and 4 weeks after the end of the 2 weeks of LT (T2).

#### Clinical assessment

Enrolled patients were further evaluated for insomnia including its type (i.e., DIS only, DMS only, or DIS and DMS), its duration, and so on. Self-report questionnaires including the Korean Version of Epworth Sleepiness Scale (KESS)^26^, Korean Version of the Geriatric Depression Scale (GDS-K)^27^, the Korean Version of Blessed Dementia Scale-Activity of Daily Living (BDS-ADL-K)^28^, and Clinical Dementia Rating Scale (CDR)^24^ were then administered to each patient at baseline. Patients with KESS score ≥ 12 were further evaluated to determine whether they would have any primary sleep disorder except insomnia disorder by a psychiatrist through a clinical interview. When these questionnaires were compared between the two groups, there was no significant difference in scores of KESS or PSQI, the distribution of insomnia type, or the duration of insomnia.

#### Outcome measures

Assessments of outcome measures were conducted at T0, T1, and T2. Assessments for AD patients included measures of subjective sleep quality using PSQI, cognitive functions using the MMSE in the Korean Version of CERAD Packet (MMSE-KC), Trail Making Test (TMT-A), Digit Span Test Forward (DSF), and Digit Span Test Backward (DSB), mood using the Korean Version of the Cornell Scale for Depression in Dementia (CSDD-K), Visual Analogue Scale (VAS)-Global Vgor (GV) and VAS-Global Affect (GA), and behavior symptoms using the Korean Version of the Neuropsychiatric Inventory Questionnaire (severity) (KNPI-Qs). Objective sleep was also assessed using sleep parameters from actigraphy (Actiwatch 2; Philips Respironics, Murrysville PA, USA) recording along with sleep diary. Caregiver burden was measured using the Korean Version of Zarit Burden Interview(ZBI-K)^29^ and Korean Version of the Neuropsychiatric Inventory Questionnaire (distress) (KNPI-Qd)^30^.

#### Actigraphical measures

The enrolled patients were made to wear the wrist actigraphy for 5 consecutive days from the start of T0, T1 and T2, respectively. Actiwatch recording was done at one-min epochs using wake-threshold value of 40 activity counts that would give the best compromise between detecting sleep and wake in terms of sensitivity and specificity compared to polysomnography^31^. Actigraphy data were derived with Actiware-Sleep Software (version 6.0.2, Philips Respironics, Murrysville, PA, USA). Quality assessment was performed for actigraphy data prior to analysis. Invalid actigraphy data were excluded due to problems such as technical errors of actiwatch software and fairly deviated sleep–wake patterns caused by special events or alcohol drinking. Data without movement and/or light signal for 1 h or more were treated as missing data. When compliance with wearing the actiwatch was problematic (i.e., no movement or light signal for 4 h/day or more), data for those days were discarded. Objective nocturnal sleep parameters were calculated based on the sleep period from light off to light on according to their sleep diaries. Sleep parameters included time in bed (TIB), total sleep time (TST), sleep onset (SO), sleep onset latency (SOL), wake time after sleep onset (WASO), sleep efficiency (SE), and fragmentation index.

#### Saliva melatonin assay

**Figure 1:**
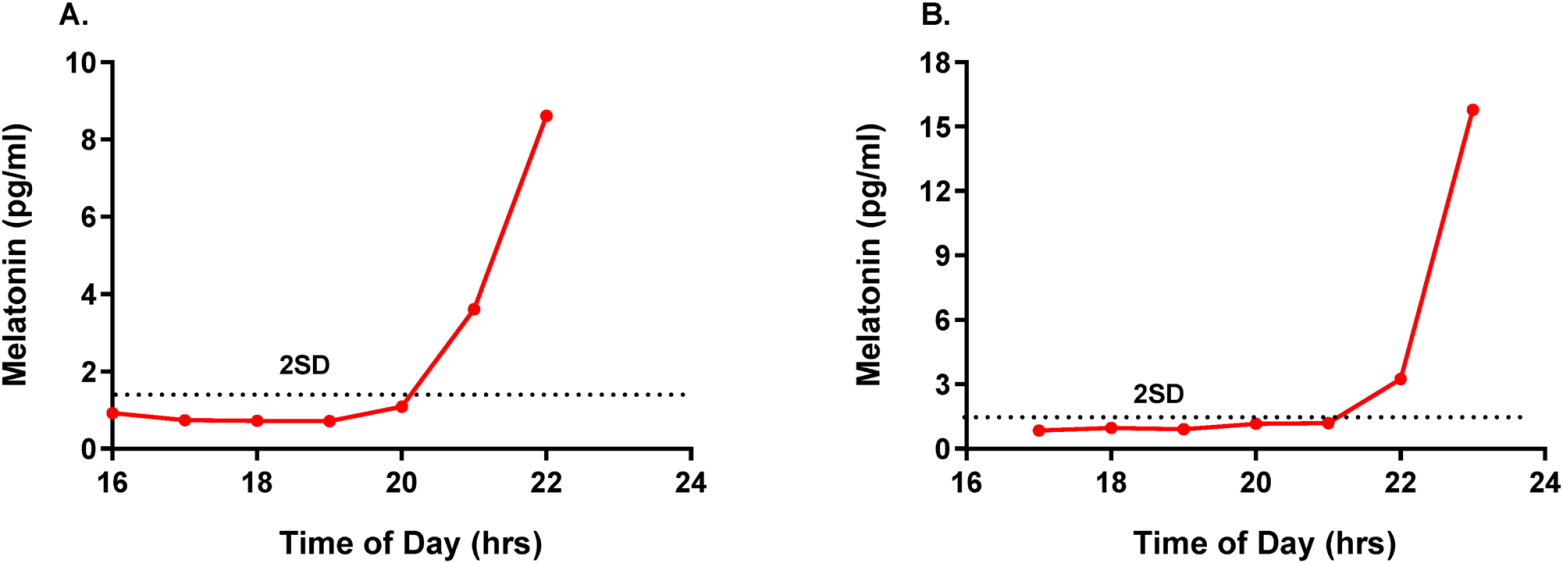
Representative salivary melatonin profiles collected under the dim light conditions in our AD patients. Dim light melatonin onset (DLMO)s were determined as a threshold calculated at 2 SD above the average baseline samples. In the presented AD patients, the DLMO was 20:01 (**A**) and 21:04 (**B**), respectively.

Participants were not allowed to take chocolate, bananas, aspirin, or analgesics on the day of the saliva melatonin assay. They were asked to visit our research laboratory, 1 h before the starting time of the saliva melatonin assay. During the saliva melatonin assay, they were made to remain where light intensity remains in dim light (~ 15 lx). If necessary, they were allowed to stay with their caregiver during the saliva melatonin assay. Seven hourly saliva samples were obtained starting from 6 h prior to sleep onset measured by actigraphy at baseline. Salivary samples were collected by the passive drool method, in which the participant allows saliva to pool in their mouth and then drools (rather than spits) through a straw into the collection tube. Each time a quantity of saliva was collected less than 2 ml. Samples were then stored in a − 20° freezer until shipped for analysis^32^. A commercially available Direct Saliva Melatonin ELISA Assay Kit (Bühlmann Laboratories AG, Switzerland) was used for the salivary melatonin assay process, assay process, according to the procedure based in the manual^33^. The assay procedure follows the basic principle of a competitive ELISA whereby there is competition between a biotinylated and a non-biotinylated antigen for a fixed number of biotinylated antigens bound to the antibody is inversely proportional to the analyte concentration of the sample^32^. Analytical sensitivity was 0.3 pg/mL^32^. The DLMOs were determined as a threshold calculated at 2 SD above the average baseline samples. There was one case as a low secretor. For its melatonin values were ranged from 1.6 to 2.3 pg/ml, we could not determine the DLMO. Thus, we assumed the DLMO as the half hour after the last sample time. Two representative salivary melatonin profiles collected under the dim light conditions in our AD patients are given in Fig. 1.

#### Timed BLT

**Figure 2:**
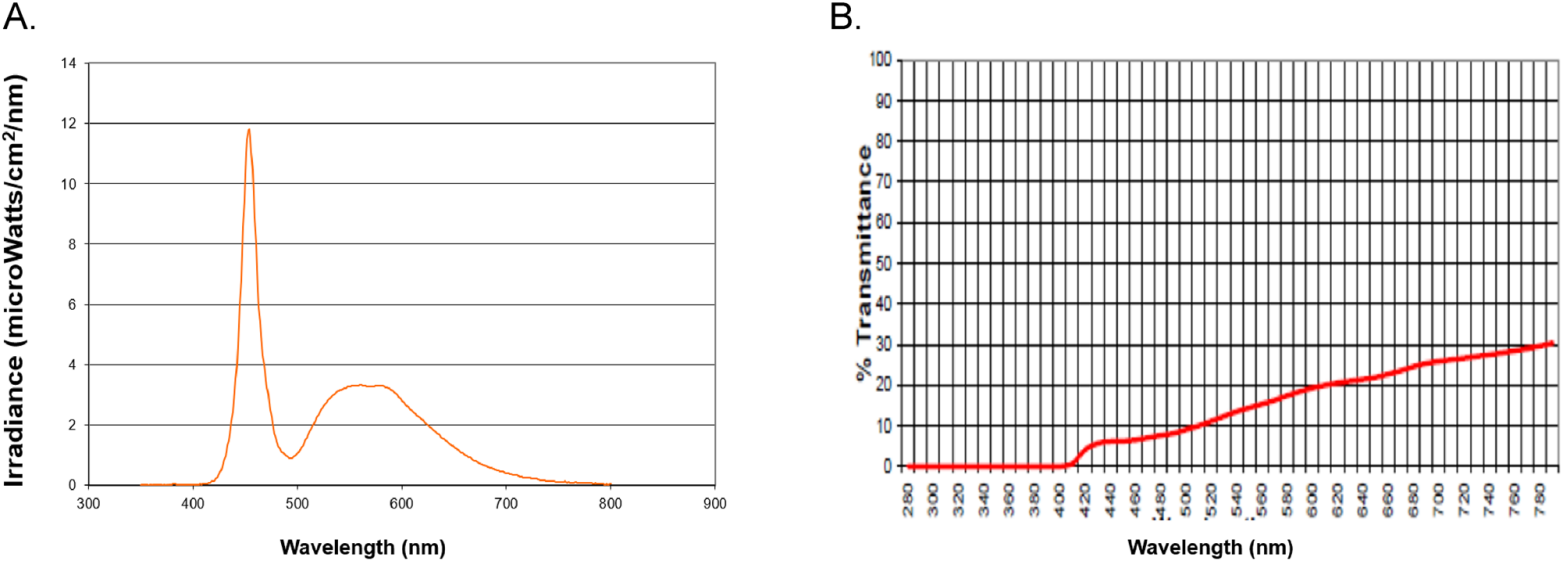
The spectral energy distributions of light emitted by the blue enriched white light treatment device (**A**) and the percentage of light transmitted through blue-attenuating glasses (**B**).

Timed BLT was administered via a Litebook treatment device with 60 light-emitting-diodes (LEDs) (Litebook Edge, 136 mm × 73 mm × 16 mm; Litebook Company Ltd., Alberta, Canada). The spectral energy distributions of light emitted by the device are shown in Fig. 2A. The 60 LEDs employed in this Litebook model contain emitters which have a spectral emission peak at approximately 464 nm and fluorescent phosphors which provide a broader, secondary spectral peak near 564 nm: of the energy emitted over the range 400–700 nm, about 48% is emitted over the range 420–508, and 37% is emitted over the range 512–616 nm. Collectively the emitted light appears white. Participants were instructed to sit about 60 cm way from the light device positioned on the table. Participants were allowed to do other activities such as reading or listening to music within 2 feet of the light source during the LT. Controls kept wearing blue-attenuating glasses during timed BLT. Percentage of light transmitted through the blue-attenuating glasses (Lightguard Hazelnut; Cocoons Live Eyewear Company, CA, USA) has been given in Fig. 2B. The transmittance of wavelengths from 410 up to 510 nm through the blue-attenuating glasses appears less than 10%. The transmittance of all the other wavelengths also is attenuated wearing the glasses, ranging between about 10 and 30%. We measured the illuminance level delivered from the device at a distance of 60 cm using a light meter (Testo 545; Testo SE & Co., PA, USA). Its illuminance levels were approximately 30 lx at the eyes without the glasses, and approximately 10 lx with the glasses. Caregivers were required to assist their patients during the timed LT and monitor their compliance to the intervention. The adherence to our protocol on the timing and duration of the timed LT was evaluated three times over the 2-week intervention by our research coordinator through phone interviews that were conducted on the first, 7th and 14th day of the intervention. There were a few patients with habitual wake time which is 1 h or more later than their scheduled LT time based on DLMO. In such cases, we made them wake up 30 min. or 1 h earlier than their habitual wake time. In addition, unwanted effects including dry eye, headache, nausea, hypomanic episode, irritability, and restlessness after beginning the timed LT were monitored through the phone interviews. Although two patients (one in each TG and CG) were made to receive the LT for at least 30 min because they had difficulty in maintaining the LT for 1 h daily due to lack of compliance, they were included in our analysis. However, there were three patients in the TG and one patient in the CG who dropped out due to behavioral disturbances during the LT, and they were excluded in our analysis.

### Measures

#### Questionnaires of outcome measures

##### Pittsburgh Sleep Quality Index (PSQI)^25^

The PSQI is an instrument to measure sleep quality in clinical populations and PSQI global scores derived by summing seven component scores ranged from 0 to 21. A patient with a global score above 5 was considered to have sleep disturbance.

##### Korean Version of the Neuropsychiatric Inventory Questionnaire (KNPI-Q)^30^

The severity score of the KNPI-Q (KNPI-Q severity, range 0–36) was used to assess the severity of a patient's behavioral symptoms. The Neuropsychiatric Inventory Questionnaire (NPI-Q) was a caregiver-based questionnaire measuring the presence and severity of 12 neuropsychiatric symptoms during the preceding month for patients with dementia (NPI-Q severity) as well as caregiver distress (NPI-Q distress).

##### Visual Analogue Scale (VAS)^34^

Subjective activation and mood were assessed by administering eight items in visual analogue scale (VAS), in which responses were indicated along 100-mm lines. Eight of these items included Global Vigor and Affect (GVA) visual analog scales validated by Monk^34^. The GVA has been shown to be sensitive to sleep loss^35^ and circadian variation^36^. GVA was calculated as follows:

$$\begin{aligned} & {\text{Global vigor }}\left( {{\text{GV}}} \right) = {{\left[ {\left( {{\text{alert}}} \right) + {3}00 - \left( {{\text{sleepy}}} \right) - \left( {{\text{effort}}} \right) - \left( {{\text{weary}}} \right)} \right]} \mathord{\left/ {\vphantom {{\left[ {\left( {{\text{alert}}} \right) + {3}00 - \left( {{\text{sleepy}}} \right) - \left( {{\text{effort}}} \right) - \left( {{\text{weary}}} \right)} \right]} 4}} \right. \kern-\nulldelimiterspace} 4}; \\ & {\text{Global affect }}\left( {{\text{GA}}} \right) = {{\left[ {\left( {{\text{happy}}} \right) + \left( {{\text{calm}}} \right) + {2}00 - \left( {{\text{sad}}} \right) - \left( {{\text{tense}}} \right)} \right]} \mathord{\left/ {\vphantom {{\left[ {\left( {{\text{happy}}} \right) + \left( {{\text{calm}}} \right) + {2}00 - \left( {{\text{sad}}} \right) - \left( {{\text{tense}}} \right)} \right]} 4}} \right. \kern-\nulldelimiterspace} 4}. \\ \end{aligned}$$

##### Korean version of the Cornell Scale for Depression in Dementia (CSDD-K)^37^

The CSDD-K was a 19-item tool designed to rate symptoms of depression for those with dementia. It had a cutoff score of 7 for depression in AD patients.

#### Caregiver burden questionnaires

##### Zarit Burden Interview (ZBI-K)^29^

Caregiver burden was measured using the ZBI-K which contained two subscales—one measuring psychological distress (termed Personal Strain, comprising 12 items), one measuring the impact of caregiving (termed Role Strain, comprising six items).

##### Korean Version of the Neuropsychiatric Inventory Questionnaire (KNPI-Q)^30^

The distress score of the KNPI-Q (KNPI-Q distress, range 0–60) was used to assess the level of caregiver distress for behavioral symptoms in patients with dementia during the preceding month.

### Statistical analysis

Demographic data, clinical data, sleep characteristics, and objective sleep parameters at baseline were compared between TG and CG using Chi-square test for categorical variables and independent t-test for continuous variables as appropriate. Data of outcome variables measured at three time points were collected, and they were compared using paired t-tests (T0 vs. T1; T0 vs. T2) in the TG and CG, respectively. Considering that some patients did not have complete dataset across three time points, generalized estimating equations analyses (GEE) were performed to examine associations between timed BLT and changes of outcome variables across three time points (T0, T1, T2). Normal as the distribution and Identity as the link function were used to estimate 95% confidence intervals. The model was fitted assuming an exchangeable correlation structure with robust standard errors. The main effects of group and time, and the interaction of Group × Time were analyzed with GEE. Outcome variables were being treated as a continuous variable in the GEE models.

All statistical analyses were performed using SPSS software package, version 18.0 (SPSS Inc., Chicago, IL, USA). Two-sided *p* values of less than 0.05 were considered statistically significant.

## Results

### Demographics data and baseline clinical characteristics

There was no significant difference in demographic data (including age, gender ratio, education level) or in clinical characteristics (including scores of ADL, GDS-K, and MMSE-KC, distribution of CDR scores) between TG and CG (Table 1).

**Table 1:** Baseline characteristics in the TG (N = 14) and CG (N = 11).

	TG	CG	*p-*value
Age (years)	77.36 ± 5.79	78.55 ± 7.71	0.664
Gender (M:F)	2:12	5:6	0.085
Education (years)	5.14 ± 3.82	5.82 ± 4.00	0.671
ADL	3.61 ± 1.67	4.55 ± 1.59	0.167
CDR (0.5:1:2)	7:5:2	5:5:1	0.856
GDS-K	11.64 ± 7.37	10.18 ± 7.61	0.632
MMSE-KC	16.36 ± 5.09	16.90 ± 4.91	0.949
DLMO	19.70 ± 1.69	20.21 ± 0.96	0.359
Start time of LT (h:m)	05:40 ± 1:09 (n = 9)	05:57 ± 0:48 (n = 5)	0.650

Independent-t test or X^2^ test, Data are shown as Mean ± SD or Ratio.

*TG* treatment group, *CG* control group, *ADL* activities of daily living, *CDR* Clinical Dementia Rating, *GDS-K* Korean version of the Geriatric Depression Scale, *MMSE-KC* MMSE in the Korean version of CERAD Packet, *DLMO* Dim light melatonin onset, *LT* light treatment.

### Changes in outcome variables across three time points

**Figure 3:**
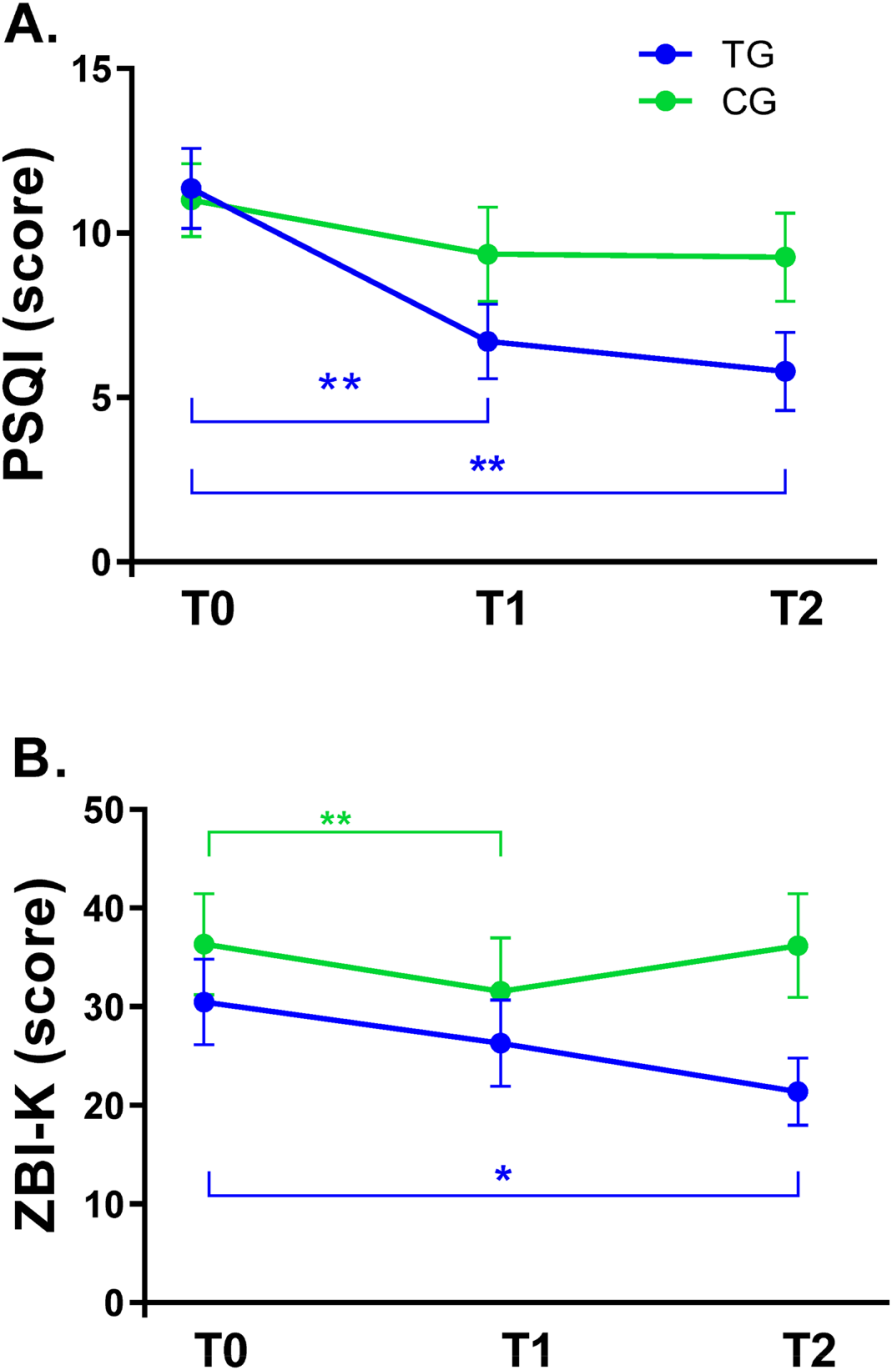
Pittsburgh Sleep Quality Index (PSQI) (**A**) and Korean version of Zarit Burden Interview (ZBI-K) (**B**) scores before and after timed blue enriched white light treatment in the treatment group (TG) (N = 14) and control group (CG) (N = 11). *T0* baseline, *T1* immediate post-treatment, *T2* 4 weeks after the end of the 2 weeks of light treatment. The mean scores for the PSQI and ZBI-K at T1 and T2 were compared to those at T0. The plots indicate mean ± SEM, *p < 0.05, **p < 0.01 (paired t-test).

Means ± standard deviation of outcome variables for TG and CG at each time point (T0, T1, and T2) are shown in Tables 2 and 3. Significant results from paired t-test are presented on Fig. 3. In paired t-tests on the PSQI score, the TG showed its significant decrease immediately post-treatment and at 4-week follow-up of the LT, compared to those at baseline (p < 0.01). In paired t-tests on the ZBI-K score, the TG showed its significant decrease at 4-week follow-up of the LT (p < 0.05), and the CG did its significant decrease immediately post-treatment (p < 0.01).

**Table 2:** Changes in subjective and objective sleep variables across three time points in the TG (N = 14) and CG (N = 11).

	TG	CG
	Mean (SD)	Mean (SD)
**Subjective sleep**		
PSQI		
T0	11.36 ± 4.57	11.00 ± 3.71
T1	6.71 ± 4.25	9.36 ± 4.78
T2	5.79 ± 4.44	9.27 ± 4.45
**Objective sleep**		
SO (h:m)		
T0	21:54 ± 1.56	22:00 ± 1.42
T1	21:51 ± 1.51	22:21 ± 1.44
T2	21:52 ± 1.28	22:17 ± 1.33
MST (h:m)		
T0	02:17 ± 1.08	02:00 ± 1.19
T1	02:13 ± 0.97	01:54 ± 1.65
T2	01:39 ± 2.09	02:01 ± 0.83
SOL (min)		
T0	22.20 ± 17.12	25.80 ± 26.30
T1	16.92 ± 12.38	34.25 ± 25.60
T2	14.88 ± 10.72	23.89 ± 19.24
TST (h:m)		
T0	07:13 ± 2.16	05:55 ± 1.34
T1	07:25 ± 1.06	05:12 ± 1.66
T2	07:24 ± 1.76	05:14 ± 1.35
SE (%)		
T0	78.69 ± 11.75	70.16 ± 7.28
T1	83.43 ± 6.19	67.42 ± 14.45
T2	80.81 ± 11.67	67.26 ± 12.36
WASO (min)		
T0	60.30 ± 35.07	86.89 ± 26.07
T1	54.29 ± 26.98	80.27 ± 30.84
T2	60.68 ± 34.03	100.38 ± 47.46
FI		
T0	35.64 ± 14.79	47.93 ± 9.66
T1	32.53 ± 12.25	50.91 ± 19.40
T2	38.54 ± 19.02	47.25 ± 18.46

*T0* baseline, *T1* immediate post-treatment, *T2* 4 weeks after the end of the 2 weeks of light treatment, *PSQI* Pittsburgh Sleep Quality Index, *SO* sleep onset, *MST* Mid-sleep time, *SOL* sleep onset latency, *TST* total sleep time, *SE* sleep efficiency, *WASO* wake after sleep onset, *FI* fragmentation index.

**Table 3:** Changes in cognition, mood and behavior across three time points in the TG (N = 14) and CG (N = 11).

	TG	CG
	Mean (SD)	Mean (SD)
**Cognitive function**		
MMSE-KC		
T0	16.36 ± 5.09	16.90 ± 4.91
T1	17.64 ± 5.68	17.91 ± 5.09
T2	17.14 ± 4.92 `	18.36 ± 5.43
TMT-A (min)		
T0	152.90 ± 63.45	98.67 ± 37.22
T1	142.25 ± 43.87	130.14 ± 85.13
T2	136.63 ± 59.98	86.33 ± 31.69
DSF		
T0	6.36 ± 2.64	7.40 ± 2.59
T1	6.64 ± 2.73	7.30 ± 2.91
T2	5.57 ± 2.77	7.50 ± 2.80
DSB		
T0	3.36 ± 2.53	4.30 ± 2.00
T1	4.43 ± 2.28	4.70 ± 2.11
T2	3.43 ± 2.28	4.60 ± 2.50
**Mood and behavior**		
CSDD-K		
T0	8.07 ± 6.03	10.00 ± 6.59
T1	5.50 ± 4.54	10.18 ± 7.68
T2	5.07 ± 3.89	8.09 ± 5.49
VAS-GV		
T0	73.16 ± 11.03	74.68 ± 2.88
T1	76.23 ± 1.36	75.55 ± 2.2
T2	75.37 ± 2.61	75.28 ± 2.52
VAS-GA		
T0	52.94 ± 1.51	52.98 ± 1.64
T1	52.20 ± 2.13	51.95 ± 2.62
T2	52.75 ± 1.56	52.51 ± 1.95
KNPI-Q(s)		
T0	8.29 ± 6.37	10.91 ± 5.28
T1	5.86 ± 5.96	9.18 ± 5.96
T2	5.58 ± 5.29	8.27 ± 7.09
**Caregiver burden**		
ZBI-K		
T0	30.46 ± 15.68	36.36 ± 17.00
T1	26.31 ± 15.82	31.55 ± 18.01
T2	21.38 ± 12.35	36.18 ± 17.39
KNPI-Q(d)		
T0	9.62 ± 10.40	12.55 ± 6.44
T1	6.08 ± 7.84	12.45 ± 9.18
T2	5.85 ± 6.53	10.36 ± 10.07

*T0* baseline, *T1* immediate post-treatment, *T2* 4 weeks after the end of the 2 weeks of light treatment, *MMSE-KC* MMSE in the Korean version of CERAD Packet, *TMT-A* trail making test-A, *DSF* digit span test forward, *DSB* digit span test backward, *CSDD-K* Korean version of the Cornell Scale for Depression in Dementia, *VAS-GV* visual analogue scale for global vigor, *VAS-GA* visual analogue scale for global affect, *KNPI-Q(s)* Korean Version of the Neuropsychiatric Inventory Questionnaire (severity), *ZBI-K* Korean version of Zarit Burden Interview, *KNPI-Q(d)* Korean Version of the Neuropsychiatric Inventory Questionnaire (distress).

### Generalized estimating equations (GEE) models for changes of outcome variables

GEE models were used to estimate changes of outcome variables at T1 and T2 with respect to T0 and differences in changes of those variables between TG and CG (Table 4).

**Table 4:** GEE models for the changes of outcome variables before and after the timed light treatments.

Outcome variables	Group effects		Time effects				Interaction effects			
			From T0 to T1		From T0 to T2		From T0 to T1		From T0 to T2	
	OR	p	OR	p	OR	p	OR	p	OR	p
**Subjective sleep**										
PSQI	1.14	0.820	0.20	0.078	0.18	0.114	0.05	0.056	0.02*	**.026**
**Objective sleep**										
SO	0.91	0.869	1.41	0.339	1.47	0.443	0.69	0.392	0.66	0.440
MST	1.32	0.530	0.90	0.726	1.01	0.771	0.99	0.986	0.49	0.255
SOL	0.03	0.684	> 10	0.376	0.53	0.935	< 0.01	0.180	< 0.01	0.480
TST	3.66	0.055	0.48	0.066	0.48	0.088	1.98	0.155	2.49	0.062
SE	> 10*	**.024**	0.07	0.489	0.03	0.277	> 10	0.122	> 10	0.187
WASO	< 0.01*	**.023**	< 0.01	0.594	> 10	0.389	> 10	0.775	< 0.01	0.453
FI	< 0.01**	**.009**	> 10	0.637	0.69	0.956	< 0.01	0.516	> 10	0.670
**Cognitive function**										
MMSE-KC	1.22	0.921	5.78	0.054	9.10*	**.018**	0.63	0.696	0.24	0.199
TMT-A	> 10	0.078	> 10	0.326	< 0.01	0.466	< 0.01	0.340	> 10	0.883
DSF	0.35	0.313	0.91	0.873	1.11	0.896	1.47	0.658	0.41	0.407
DSB	0.39	0.287	1.49	0.548	1.35	0.665	0.72	0.669	0.80	0.788
**Mood and behavior**										
CSDD-K			1.20	0.915	0.15	0.292	0.06	0.215	0.34	0.646
VAS-GV	0.23	0.627	2.37	0.074	1.83	0.311	9.58	0.431	5.28	0.550
VAS-GA	1.06	0.923	0.36	0.163	0.63	0.323	1.47	0.669	1.33	0.649
KNPI-Q(s)			0.18	0.218	0.07	0.054	0.50	0.752	0.93	0.970
**Caregiver burden**										
ZBI-K	< 0.01	0.359	0.02	0.068	< 0.01*	**.018**	0.52	0.802	< 0.01	0.081
KNPI-Q(d)	0.03	0.280	0.03	0.187	0.02	0.180	> 10	0.286	4.89	0.657

T0 = baseline, T1 = immediate post-treatment, T2 = 4 weeks after the end of the 2 weeks of light treatment. [T0] as reference for the Time effects; [CG] and [T0] as reference for the Interaction effects.

*GEE* generalized estimating equations, *OR* odd ratio, *TG* treatment group, *CG* control group, *PSQI* Pittsburgh Sleep Quality Index, *SO* sleep onset, *MST* Mid-sleep time, *SOL* Sleep onset latency, *TST* Total sleep time, *SE* Sleep efficiency, *WASO* Wake after sleep onset, *FI* Fragmentation index, *MMSE-KC* MMSE in the Korean version of CERAD Packet, *TMT-A* Trail making test-A, *DSF* Digit span test forward, *DSB* Digit span test backward, *CSDD-K* Korean version of the Cornell Scale for Depression in Dementia, *VAS-GV* Visual analogue scale for global vigor, *VAS-GA* Visual analogue scale for global affect, *KNPI-Q(s)* Korean Version of the Neuropsychiatric Inventory Questionnaire (severity), *ZBI-K* Korean version of Zarit Burden Interview, *KNPI-Q(d)* Korean Version of the Neuropsychiatric Inventory Questionnaire (distress).

*p < 0.05, **p < 0.01.

Regarding subjective sleep quality, there was no significant main effect of time on PSQI scores at either T1 or T2 with respect to T0. However, there was a significant group-by-time interaction on PSQI scores at T2 with respect to T0, indicating that a reduction of PSQI score from baseline to 4-week follow-up of the LT in TG was significantly greater than that in CG (OR: 0.02, *p* = 0.026). Regarding objective sleep, there was no significant main effect of time on objective sleep parameters at either T1 or T2 with respect to T0 while there was significant main effect of group on SE, WASO and FI (p < 0.05 or 0.01). There was no significant group-by-time interaction on objective sleep parameters, indicating no significant effect of timed BLT on TG or CG. Regarding cognitive functions, there was a significant main effect of time on MMSE-KC scores at T2 with respect to T0 (OR: 9.10, *p* = 0.018), indicating a significant change of MMSE-KC score from baseline to 4-week follow-up of the LT. Regarding mood and behavior symptoms, there was no significant main effect of time on scores of CSDD-K, VAS-GV, VAS-GA, or KNPI-Q(s) at either T1 or T2 with respect to T0. There was no significant group-by-time interaction on these scores either, indicating no significant effect of timed BLT on TG or CG. Regarding caregiver burden, there was a significant main effect of time on ZBI-K scores at T2 with respect to T0 (OR ≤ 0.01, *p* = 0.018), indicating a significant change in ZBI-K score from baseline to 4-week follow-up of the LT.

## Discussion

The objective of this study was to determine whether timed BLT could improve sleep, cognition, mood, and behavior symptoms of home-dwelling patients with mild and moderate degree of AD and whether it could relieve the burden of their caregivers as a consequence of improving these outcomes. In a single-blind, placebo-controlled study with a between-within design, it was hypothesized that timed BLT could beneficially improve these outcome measurements in AD patients more than timed blue-attenuating LT as a control condition. It was also hypothesized that changes in these outcomes immediate post-treatment and at 4-week follow-up of the LT compared to baseline would depend on timed light interventions.

We found significantly better effectiveness of timed BLT on subjective sleep quality, measured by PSQI scores in comparison with that of timed blue-attenuating LT (OR: 0.02, *p* = 0.026). However, there was no significant change in PSQI scores after timed light interventions compared to those at baseline (Table 4). In subsequent analyses, we also found that PSQI scores in TG were significantly reduced at both immediate post-treatment and 4-week follow-up of the LT compared to those at baseline (paired t-test, p < 0.01), but not in CG (Fig. 3A). Our findings may indicate that timed BLT has immediate and lasting effects on subjective sleep quality in patients with mild and moderate AD. However, only this lasting effect was superior to that of timed blue-attenuating LT. These inconsistent findings regarding the immediate and lasting effects on subjective sleep quality could reflect likelihood of type 2 errors that might be due to the relatively small sample size. Our findings were similar to those of Sloane et al.^2^, who showed that PSQI component scores of sleep efficiency were improved after the intervention using a blue-white light box (15 × 15 cm) in home-dwelling patients, although they did not find a differential effect between blue-white light and a red-yellow light as control intervention. Figueiro et al.^16,38^ have also observed a significant decrease in PSQI global score after the light intervention in institutionalized AD patients using a blue-white light fixture (24 × 7 × 7.5 inches). Simultaneously, they^16^ showed that PSQI scores were still lower at 4 weeks after the lighting intervention, suggesting its lasting effect on subjective sleep quality. The effect of light intervention on subjective sleep would be different depending on the size of applied light boxes. Meanwhile, lifestyle regularity (i.e., regular sleep–wake habits, regular exercise) itself may function as a zeitgeber. It can improve sleep quality^39^. Thus, our finding may result from a consequence of regularity in their wake times obtained over consecutive 2 weeks of LT, suggesting a coexisting possibility that short wavelength light can help AD patients keep a good sleep quality. Given our light intervention based on individual circadian phase, their improved sleep quality after the timed BLT should be considered in relation to its phase-shifting effects on the circadian system. Thus, further studies are required to investigate the relationship between the changes in circadian phase and those in sleep quality after the timed BLT in those patients. Only three of our patients had later DLMOs than the healthy older subjects in a previous study^40^. In that study the DLMOs ranged from 17.4 to 21.6 h. The three latest DLMOs in our patients were 21.7, 22.0 and 22.1 h. Thus, the DLMOs in our AD patients were not unusually late. Results of previous studies on the effectiveness of LT, regardless of its lighting source (i.e., blue or bright light) or dementia severity, on objective sleep of dementia patients have been inconsistent. Figueiro et al.^16^ using a blue-white light fixture have found improvements in TST and SE measured by actigraphy in mostly severe patients (MMSE score of 7.7 ± 2.3). However, Sloane et al.^2^ using a blue-white light box have shown no effect of blue-white light intervention on sleep parameters of home-dwelling patients (MMSE score of 12.7 ± 9.1). In the same vein, bright LT increased sleep duration of AD patients in a long-term study by McCurry et al.^41^ using a broad spectrum fluorescent light box, but not in other studies^3,9^. These inconsistent findings suggest that setting the timing of LT without considering individual circadian phase might be inappropriate. Although there was no significant change in objective sleep outcomes after timed light interventions compared to those at baseline, we found a trend in effectiveness of timed BLT on TST at 4-week follow-up of the LT with respect to baseline in comparison with that of timed blue-attenuating LT (OR: 2.49, *p* = 0.062), indicating a beneficial delayed effect of timed BLT on objective sleep duration (Table 4). Otherwise, it has been suggested that long-term light exposure can provide a stabilizing effect on the circadian system, leading to sleep consolidation^7,12,42^. Thus, small or non-significant changes of objective sleep parameters in our study might be somewhat linked to the duration of LT because 2 weeks might be relatively short. Nevertheless, since this is the first study that determines LT timing based on individual circadian phase, our findings need to be verified by further studies.

We found no differential effectiveness of timed BLT on MMSE-KC, TMT-A, DSF, or DSB scores in comparison with that of timed blue-attenuating LT. However, there was a significant increase in MMSE-KC score at the 4-week follow-up after timed light intervention compared to that at baseline (time effect, OR: 9.10, *p* = 0.018), together with a tendency of increase in its score at immediate post-treatment (time effect, OR: 5.78, *p* = 0.054) (Table 4). Our findings suggest that timed light intervention itself regardless of the presence of short wavelength light can improve global cognitive function of the AD patients, having a lasting effect. Conflicting findings have been found in previous studies regarding the effectiveness of LT on cognitive function in dementia patients^3,12^. Such conflicting results are attributable to heterogeneity in study populations, especially for those with institutionalized setting. Given the response to light can vary according to the type of dementia^17^, our findings for a homogenous group of AD patients would be distinct from those of previous studies. A study has shown that exposure to the most blue enriched light source the most blue enriched white light source among 3 different lights was most effective in enhancing cognitive performance for the tasks of sustained attention^43^. It has been known that blue light has an impact on cognitive performance via both its alerting effect and circadian phase-shifting effect^42^. A recent functional magnetic resonance imaging study implicates that these effects of blue light might modify the activity of brain areas, especially those involved in sustained attention^44^ rather than executive function. However, we found no evidence of an effect of timed BLT on the domains of attention and executive function measured by DST and TMT, respectively. Particularly, the performance of these cognitive domains is more likely to be affected by the time of the day^45,46^. In our study, three-time evaluations required for each cognitive test were not done at a certain time of the day. Thus, the time of the day effect on cognitive performance might have affected our non-significant finding.

In mood symptoms, we found no differential effectiveness of timed BLT on CSDD-K or VAS scores in comparison with that of timed blue-attenuating LT. There were no significant changes in scores of those scales after timed light interventions either (Table 4). Somewhat differently from our findings, in a recent study by Figueiro et al.^36^, a lighting intervention tailored to maximally affect the circadian system showed the significantly improved CSDD scores compared to the baseline. Likewise, Sloane^2^ has shown significant improvements in depressive symptoms measured by the CSDD scores after a blue-white light intervention in home-dwelling dementia patients, although they have failed to find significantly superior effect on symptoms in comparison with the control condition. Given that CSDD-K cut-off score of 7 has been identified for depression in AD patients^37^, our TG showing a mean score of 8 might indicate a relatively low degree of their depressive symptoms. Thus, we need to consider the possibility of its floor effect for TG which might have contributed to our non-significant finding of depressive symptoms. Another possibility is that mood symptoms of our patients might not be sensitively reflected using VAS, since VAS is useful for assessing a change in diurnal mood rather than mood symptom itself^33^. Neuropsychiatric symptoms such as aggressiveness, agitation, and disorganized behavior seem to be more frequent in advanced stage of dementia than in its earlier stage^47^. However, in previous studies using broad spectrum fluorescent light boxes for severe AD patients, only non-significant or small changes were found in their neuropsychiatric behavior symptoms after LT^3,12,48^. We found that there was a tendency of reduction in KNPI-Q(S) scores at 4-week follow-up after timed light interventions compared to those at baseline (time effect, OR: 0.07, *p* = 0.054), though it did not reach statistical significance (Table 4). Taken together, our finding might only reflect responses of neuropsychiatric symptoms to LT in our study subjects with relatively earlier stage of dementia.

In the aspect of caregiver burden, we found no differential effectiveness of timed BLT on ZBI-K scores in comparison with that of timed blue-attenuating LT. However, there were significant reductions in ZBI-K scores at 4-week follow-up after timed light interventions compared to those at baseline (time effect, OR < 0.01, *p* = 0.018), indicating a delayed effect of timed LT regardless of its lighting source on caregiver burden (Table 4). In subsequent paired t-tests, we found that ZBI-K scores in TG were significantly reduced at 4-week follow-up of the LT compared to those at baseline (p < 0.05), while its scores in CG were significantly reduced immediate post-treatment (p < 0.01) (Fig. 3B). Given the significant decrease of ZBI-K scores at 4-week follow-up of the LT observed only in the TG, it is likely to have a delayed effect of timed BLT on caregiver burden. On the other hand, Sloane et al.^2^ have shown that there are no differential effects of blue-white light intervention on ZBI scores in comparison with control intervention, although scores of role strain in ZBI are significantly reduced during intervention relative to baseline. In our study, differently from the finding on ZBI-K, there were no significant changes in KNPI-Q(d) scores reported by caregivers either immediate post-treatment or at 4-week follow-up after timed light interventions. Neuropsychiatric symptoms would exhibit a prominent feature in advanced stage of dementia^47^. Given the nature of our study subjects, the extent of behavior symptoms in our AD patients might not have been severe enough to considerably distress their caregiver.

To the best of our knowledge, this is the first study to investigate effects of timed blue-enriched white light treatment given based on individual circadian phase on outcomes of subjective and objective sleep, cognitive function, mood, and behavioral symptoms of home-dwelling patients with mild and moderate AD. Our study has some limitations. Firstly, in order to verify the hypothesis that timed BLT would have a therapeutic effect by stabilizing circadian rhythms, changes of DLMO before and after timed BLT should be estimated. However, DLMO assessment after LT could not be done due to a limited tolerability of our AD patients. Secondly, we should have controlled the time of day effect on the performance of cognitive tests. Together, we might have applied cognitive tests that are more sensitive to blue-enriched white light exposure, i.e., psychomotor ability^43^. Thirdly, since the accuracy of timed LT on assigned time could influence its outcomes, its adherence should have been monitored in an objective way, not by depending on only caregiver’s report. Lastly, our portable light box can easily be accidentally moved and point away from the eyes. Despite these limitations, our study suggests that timed BLT for 2 weeks could improve subjective sleep quality and global cognitive function of outpatients with mild and moderate AD.

In conclusion, our findings give an evidence that timed BLT could affect both subjective and objective sleep in patients with mild and moderate AD, showing its lasting effect in reducing PSQI scores and its delayed effect in increasing TST in a placebo-controlled study. Our findings also implicate that global cognitive function and behavioral symptoms of AD patients could be improved by timed light interventions. Therefore, our study suggests that timed BLT based on individual circadian phase may provide a therapeutic rationale for enhancing sleep quality and cognitive performance of AD patients in clinical setting.
